# Assessment of cell free mitochondrial DNA as a biomarker of disease severity in different viral infections

**DOI:** 10.12669/pjms.36.5.2476

**Published:** 2020

**Authors:** Zain Ali, Shahid Waseem, Riffat Aysha Anis, Mariam Anees

**Affiliations:** 1 Zain Ali, MPhil. Department of Biochemistry, Quaid-i-Azam University, Islamabad, Pakistan; 2 Shahid Waseem, Ph.D. Alpha Genomics Private Limited, Islamabad, Pakistan; 3 Riffat Aysha Anis, Ph.D. Institute of Diet and Nutritional Sciences, The University of Lahore, Islamabad Campus, Pakistan; 4 Mariam Anees, Ph.D. Department of Biochemistry, Quaid-i-Azam University, Islamabad, Pakistan

**Keywords:** HIV, HBV, HCV, Mitochondrial DNA, Interleukin-6

## Abstract

**Objective:**

Cell Free mitochondrial DNA (CF mt-DNA) has emerged as a novel biomarker to investigate disease pathophysiology of different infections. The present study was designed to elucidate the association between CF mt-DNA, IL-6 and viral load in HIV, HBV and HCV infections and predict its role as a potential biomarker to assess the disease severity in viral infections.

**Methods:**

Total 120 blood samples were collected from January 2018 to December 2018 of HIV, HBV and HCV patients and healthy controls (30 samples in each group). DNA and RNA were extracted from the serum to determine the levels of CF mt-DNA and viral load, respectively. IL-6 from the serum of infected individuals was quantified with ELISA.

**Results:**

HCV patients showed the highest levels of CF mt-DNA, IL-6 and viral load, followed by HBV and HIV. Significant correlation was found between CF mt-DNA and IL-6 among the HBV patients (p=0.017). However, no significant correlation of CF mt-DNA was observed with IL-6 in HIV and HCV or with the viral load in any of the three infections.

**Conclusion:**

Elevated CF mt-DNA indicates its role in severity of viral infections. Independence of CF mt-DNA expression from viral load and IL-6 in case of HIV and HCV suggests involvement of other inflammatory pathways regulating CF mt-DNA elevation.

## INTRODUCTION

Assessment of inflammation at molecular level is a hallmark of disease progression studies. Mitochondria plays an important role in cellular homeostasis and inflammatory response in viral infection.^1^ Mitochondria shares genetic homology with prokaryotes and the recognition of mitochondrial genome in extracellular environment by immune system initiates an inflammatory response.^2^ Role of mitochondria in damage associated molecular patterns (DAMPs) is well established. Role of Cell free mitochondrial DNA (CF mt-DNA) in disease physiology is an emerging field in the study of new biomarkers but it still need exploration.^3^ In case of infections, inflammatory response by the cells respond by increasing different biomarkers i.e. C-Reactive Protein (CRP), IL-6, TNF-alpha and many others.^4^ CF mt-DNA has shown induction of TNF-alpha and IL-6 in murine models.^5^ Elevation of IL-6 many folds in different pathophysiological conditions is well documented.^6^ Viral load in case of HIV, HBV and HCV represents the replication potential of virus and disease progression to a certain extent. Viral load is predominantly first choice as a diagnostic parameter, but it may diminish within few weeks of infection.^7^ The need to explore biomarkers to serve the purpose of diagnosis as well as disease progression and severity requires rigorous exploring.

The present study investigates the correlation of elevated CF mt-DNA levels with IL-6 and viral load in viral infections i.e. HIV, HBV and HCV and the possibility of a potential role for CF mt-DNA as a marker for disease severity and disease progression in viral infections.

## METHODS

### Sample Collection

Before the commencement of study, Ethical Approval was obtained from the Bio-Ethical Committee of Quaid-i-Azam University, Islamabad (BEC-FBS-QAU2018-74, Dated: 18-01-18). Individuals (n=30) with HIV infection were recruited from HIV treatment centers at Pakistan Institute of Medical Sciences (PIMS), Islamabad and Peshawar. Clinical information was recorded on a structured questionnaire. HBV and HCV infected serum samples (n=30 each) were collected from diagnostic centers in Islamabad. Healthy controls (n=30) were also included in the study. All the samples we acquired in the span of January 2018 to December 2018. Written informed consent was obtained from the subjects for collection and utilization of their samples for research purpose. Age, region and gender of the participants recruited in this study are provided in Table-I.

**Table-I:** Clinical parameters of HIV, HBV and HCV infected individuals along with healthy controls.

	HIV	HBV	HCV	Control
No of Patients (n)	30	30	30	30
Male (%)	73.3	66.7	100	53.3
Female (%)	26.7	33.3	0	46.7
Mean age ± SD	33.167 ± 5.546	29.867 ± 12.719	30.300 ± 12.531	28.168 ± 5.552
Age range	25 - 45	06 – 55	06 - 57	19 - 44
Median age	32	28	29	27
Punjab province (%)	46.7	33.3	30.0	33.3
AJK province (%)	13.3	26.7	30.0	13.3
KPK province (%)	40.0	40.0	40.0	53.3

The patients did not show any clinical signs of co-infection or presence of other diseases including liver cirrhosis and cancer.

### Extraction of nucleic acids for viral load estimation

Nucleic acids from serum samples of HIV, HBV and HCV infected individuals and healthy controls were extracted using a commercial kit (Machery-Nagel, Germany) according to the manufacturer’s protocol. Extracted nucleic acids were stored at -20°C until further use.

### Viral load estimation

Viremia in serum samples of HIV, HBV and HCV infected individuals was analyzed on Magnetic Induction Cycler (MIC) qPCR (BMS, Australia). Serum samples (HIV, HBV and HCV) were acquired on FAM channel while internal controls, provided in the kit, were detected on HEX channel for HBV and HCV, and on ROX channel for HIV (GeneProof and QIAGEN, Germany). Standards were included in the run to generate standard curve. Viral copies were quantified using the standard curve.

### DNA extraction for CF mt-DNA analysis

DNA was extracted from 600µl serum of HBV and HCV infected as well as healthy individuals (control) by using organic method with slight modifications.^8^ Serum and plasma both were used in case of HIV patients. DNA was eluted in 80µl of distilled water and stored at -20°C until further use.

### Reference and target genes for qPCR

Primers were used to amplify regions from mitochondrial and human genomic DNA as reported earlier.^9^ Human beta2 microglobulin (hB2M) was used as a reference to determine the fold expression of CF mt-DNA in healthy control and patient samples. hB2M primers used were hB2M F1: TGTTCCTGCTGGGTAGCTCT and hB2M R1: CCTCCATGATGCTGCTTACA. Sequence to amplify mitochondrial DNA was hmito F3: CACTTTCCACACAGACATCA and hmito R3: TGGTTAGGCTGGTGTTAGGG. Levels of CF mt-DNA were calculated with reference to the hB2M gene of cell free nuclear DNA in the serum of same samples.

### Quantification of CF mt-DNA

Freely circulating mitochondrial and nuclear DNA contents in plasma and serum were quantified by qPCR (Magnetic Induction Cycler; BMS, Australia). Each run was monitored by a negative (no template) and a positive control. All the analyses were performed in triplicate. Real-time PCR was carried out using Eva green qPCR Master mix (Solis Biodyne, Estonia). Fold expression of CF mt-DNA was assessed by amplifying a unique mitochondrial fragment relative to hB2M gene as reference. CF mt-DNA fold expression was analyzed by Pfaffle method.^10^

### ELISA of IL-6

IL-6 was quantified in serum samples of HIV, HBV and HCV infected individuals and healthy controls by IL-6 ELISA detection kit (Solar Bio, China) according to the manufacturer’s protocol. Absorbance was measured at 450 nm on Multi-Skan Go (ThermoFisher Scientific, USA).

### Statistical Analysis

Descriptive statistics were computed as mean ± SEM along with range using Statistical Package for Social Sciences (SPSS) version 22. Difference of CF mt-DNA in serum and plasma was calculated by t-test. Means of CF mt-DNA, IL-6 and viral load were compared using Analysis of Variance (ANOVA). Means of groups were further separated using Tukey’s post-hoc test. Correlations among various parameters were drawn using the Spearman Rho correlation coefficient. A p value of <0.05 was considered significant and <0.001 was taken as highly significant.

## RESULTS

### Expression of CF mt-DNA in serum of HIV, HBV and HCV infected individuals

**Fig.1:**
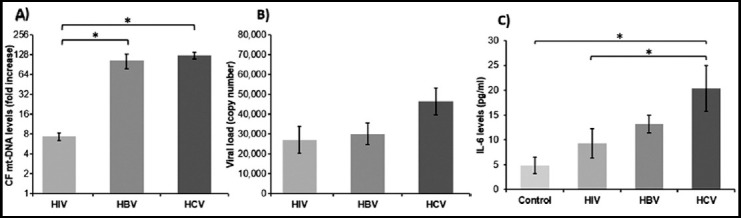
Comparative levels of CF mt-DNA, viral load and IL-6 in serum samples of HIV, HBV and HCV infected individuals. A) Fold-increase in CF mt-DNA levels as compared to healthy controls. B) Viral load comparison among the disease groups. C) Expression levels of IL-6 in the serum samples of infected individuals along with healthy controls. The * symbol above bars represents significant difference among groups (p<0.05).

CF mt-DNA levels were calculated as ‘fold increase’ in comparison to healthy controls by real time PCR. A significant elevation of CF mt-DNA represented as mean ± SEM was observed in the serum of HIV (7.412 ± 1.023), HBV (104.900 ± 26.335) and HCV patients (125.000 ± 13.391) as depicted in Fig.1A and Table-II. Presence of CF mt-DNA was significantly higher in HBV (*p*<0.001) and HCV (*p*<0.001) samples as compared to HIV as determined by One-way ANOVA and Tukey’s post-hoc tests. There was no significant difference between the expression levels of CF mt-DNA in HBV and HCV patients (*p*=0.684).

**Table-II:** Levels of CF mt-DNA, viral load and IL-6 in serum samples of HIV, HBV and HCV infected individuals.

Infection	CF mt-DNA (fold increase)	Viral Load (copy number)	Interleukin 6 (pg/ml)
HIV	7.412 ± 1.023^a^ 7.0^b^(1-34)^c^	27,218.182 ± 6,736.226 17,000 (5,700-150,000)	9.320 ± 2.897 5 (0.20-87)
HBV	104.900 ± 26.335 91.0 (3-769)	30,219.6667 ± 5,449.475 17,500 (1,000-98,000)	13.153 ± 1.765 13 (0.20-58)
HCV	125.000 ± 13.391 145.0 (12-267)	46,584.500 ± 6,824.206 45,339 (1,200-98,000)	20.367 ± 4.536 22 (0.10-141)

a Each value represents mean ± SEM.

b Each value represents median.

c Values in parenthesis represent the range of parameters.

### Quantification of viral load in serum of HIV, HBV and HCV infected individuals

Viral loads were determined in all the three infections. Their comparison exhibited highest viral copy number for HCV represented as mean ± SEM (4.658 x 10^4^ ± 0.682 x 10^4^) followed by HBV (3.022 x 10^4^ ± 0.545 x 10^4^) and HIV (2.722 x 10^4^ ± 0.674 x 10^4^) as shown in Fig.1B and Table-II. High viral loads mean either the infection is untreated, or the body is not responding to the therapy. Although a trend was observed among the viral loads of HIV, HBV and HCV; however, the differences among the three groups were not statistically significant as determined by ANOVA probably due to high variance.

### Measurement of IL-6 in serum of HIV, HBV and HCV infected individuals

IL-6 levels were measured in the serum of healthy controls as well as HIV, HBV and HCV infected individuals. Lowest IL-6 levels were observed in the healthy controls represented as mean ± SEM (4.833 ± 1.647 pg/ml) and a gradual increase was witnessed in HIV (9.320 ± 2.897), HBV (13.153 ± 1.765) and HCV (20.367 ± 4.536) infected individuals (Fig.1C and Table-II). Difference of IL-6 expression remained non-significant between HIV and HBV patients (*p*=0.795) and HBV and HCV patients (*p*=0.313); however, the difference between HIV and HCV patients was statistically significant (*p*=0.045). The control group IL-6 levels were lower than all infected groups but significantly lower than HCV patients only (*p*=0.002).

### Comparative expression of CF mt-DNA in HIV serum and plasma

**Fig.2:**
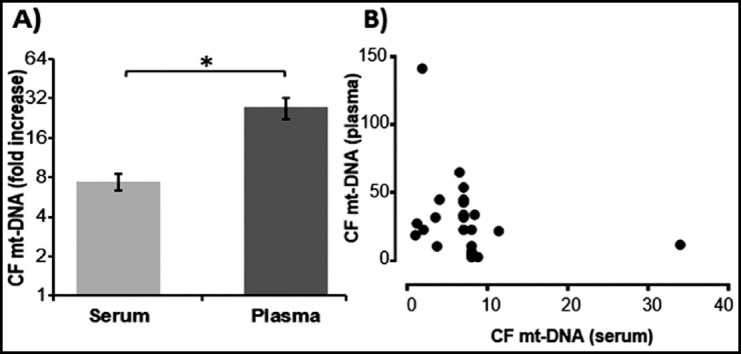
Comparative analysis of CF mt-DNA in serum and plasma samples of HIV infected individuals. A) Expression of CF mt-DNA was higher in plasma as compared to serum. The * symbol above bars represents significant difference among groups (p<0.05). B) No significant correlation was observed between the serum and plasma concentration of CF mt-DNA in HIV samples.

Levels of CF mt-DNA in plasma and serum samples of HIV infected individuals were calculated. Comparative analysis showed significantly higher (*p*<0.0001) levels of CF mt-DNA in plasma (27.309 ± 5.029) of HIV infected individuals than their serum samples (7.413 ± 1.024) as shown in Fig.2A. However, both the values were independent of each other and the data did not indicate any significant correlation of CF mt-DNA in the serum and plasma samples of HIV infected individuals (Fig.2B).

### Correlation between viral load and CF mt-DNA in serum samples of HIV, HBV and HCV

**Fig.3:**
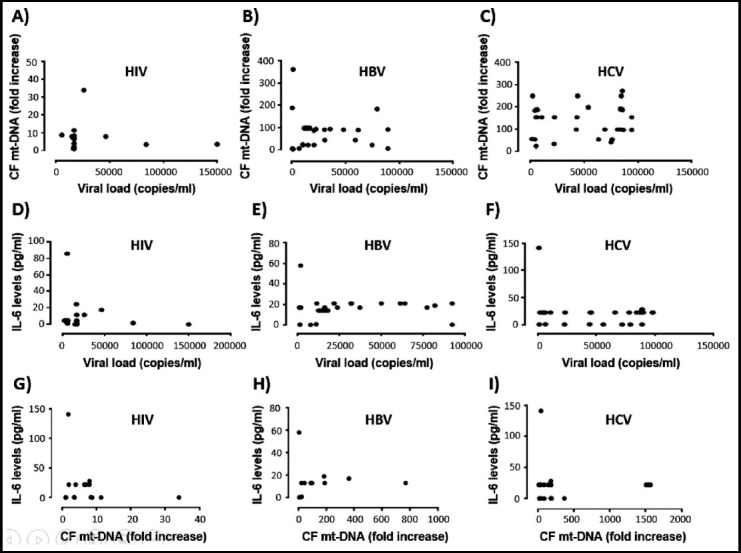
Spearman correlations between CF mt-DNA, viral load and IL-6 among the three disease groups. A-C) Correlations between viral load and CF-mt DNA in blood serum of HIV, HBV and HCV infected individuals. No significant correlation was observed between the two entities in any of the viral infections. D-F) Correlation between CF mt-DNA and IL-6 in blood serum of HIV, HBV and HCV infected individuals. A significant correlation was observed between the two entities only in HBV patients. G-I) Correlation between viral load and IL-6 in blood serum of HIV, HBV and HCV infected individuals. No significant correlation was observed between the two entities in any of the viral infections.

Spearman Rho correlation coefficient was used to see the relationship between CF mt-DNA and viral load. The viral load and CF mt-DNA both were elevated in infected individuals as compared to healthy controls; however, there existed no significant correlation between viral load and CF mt-DNA in serum of HIV, HBV and HCV infected persons as shown in Fig.3A-C and Table-III. Both the entities of viral load and CF mt-DNA were independent of each other.

**Table-III:** Spearman Rho correlations among the CF mt-DNA, IL-6 and viral load in three infectious groups.

Variables	HIV		HBV		HCV	

	CF mt-DNA	IL-6	CF mt-DNA	IL-6	CF mt-DNA	IL-6
Interleukin 6	0.186^a^		0.432		0.056	
	0.407^b^		0.017*		0.768	
Viral Load	-0.071	0.163	-0.234	0.135	0.144	0.034
	0.755	0.496	0.213	0.477	0.449	0.858

a Each value represents the value of Spearman Rho correlation coefficient (R).

b Each value represents the level of significance (p).

* Significant correlation (p<0.05).

### Correlation between CF mt-DNA and IL-6 in serum samples of HIV, HBV and HCV

Presence of CF mt-DNA was also correlated with the inflammation marker IL-6. Despite of observing high CF mt-DNA and IL-6 in all the three infections as compared to healthy controls, a direct significant correlation was observed between the two entities only in HBV patients (R=0.432; *p*=0.017). No significant correlation was seen between CF mt-DNA and IL-6 in HIV and HCV infected individuals as depicted in Fig.3D-F and Table-III.

### Correlation between viral load and IL-6 in serum samples of HIV, HBV and HCV

Viral load is naturally higher in viral infections and we saw high copy numbers in all the three infections. IL-6 levels were also higher in HIV, HBV and HCV as compared to healthy controls. However, no correlation of viral load and IL-6 was found in serum of HIV, HBV and HCV infected individuals as given in Fig.3G-I and Table-III.

## DISCUSSION

HIV, HBV and HCV infections are a serious health burden in Pakistan.^11^–^14^ Diagnosis and point of care strategies require thorough understanding of infection’s severity in the Pakistani population. Elevated CF mt-DNA levels have been observed in different pathophysiological conditions.^15^ This role of CF mt-DNA in different diseases make it an ideal candidate for its assessment in viral pathophysiology.

This study is the first to report the comparative analysis of CF mt-DNA in the serum of HIV, HBV and HCV infected individuals from Pakistan. We found higher CF mt-DNA levels in all infected individuals. A significant correlation was observed between CF mt-DNA and IL-6 in HBV infection while no such correlation was observed in HIV and HCV infections. Viral load was not significantly correlated to CF mt-DNA in any of the studied infections.

Elevated levels of CF mt-DNA in HCV infection is related to chronic and acute phases with progression into Hepatocellular carcinoma.^16^ Platelet activation is generally associated with elevated CF mt-DNA levels in serum.^17^ Platelet activation is increased in viral infections i.e. HIV and HBV which in turns is responsible for elevated CF mt-DNA levels.^18^ In case of HIV infection, integration of viral RNA into the host genome does not dictate the cell damage as it is evident in HBV and HCV infection. HIV induces apoptosis in the infected CD4 cells.^19^ During apoptosis, cellular contents are visualized, preventing CF mt-DNA to leach into the serum which may be a possible reason for less CF mt-DNA levels in serum of HIV than HBV and HCV in the present study. CF mt-DNA levels in plasma of HIV were higher than any other infection’s serum CF mt-DNA levels. Plasma contains cellular artifacts and remnants of platelets mitochondrial content which can be reason for higher CF mt-DNA levels.

IL-6 is generally known to play a dual role in inflammation by acting as anti-inflammatory cytokine through classic signaling and pro-inflammatory cytokine through trans-signaling. In the present study, IL-6 levels were also elevated in HIV, HBV and HCV infected individuals in comparison to healthy controls. IL-6 levels in HBV were elevated possibly due to suppressed antigen processing by Kupffer cells as reported earlier.^20^ Levels of IL-6 in HCV infected individuals have been reported to increase in chronic cases, prior to IFN-Gamma therapy and in non-responders to therapy.^21^ Serum and plasma levels of IL-6 also rise in HIV infection because of its primary role in the increased differentiation of polyclonal beta cells.^22^

HCV infected individuals exhibited higher levels of IL-6 in the serum than HBV and HIV. IL-6 is produced in response to infection and its levels are increased in most of the infections.^23^ HCV infection induces the production of IL-6 through the enhanced expression of Toll Like Receptor 4 (TLR-4).^24^ Higher IL-6 levels also indicate liver cirrhosis and liver fibrosis in chronic cases of HCV infection.^25^ While in case of HBV infection, increased replication of HBV decreases the levels of IL-6 due to the unabated nucleo-capsid formation.^26^ Increased levels of IL-6 in HCV infected individuals and decreased levels in HBV infected individuals both suggest the severity of these infections independent of their viral load, while IL-6 levels in HIV infected individuals suggest lower virulence of HIV among the infected individuals. No causal relationship between viral load and IL-6 levels has been reported earlier.^27^

### Limitations of the study

Only patients with mono-infection were recruited for this study. Other infections i.e. HCV, HBV and TB which are commonly associated with HIV infection among Pakistani population are not represented in this study. Also, all the patients in this study were therapy-naïve. To further elucidate the role of CF mt-DNA as a biomarker for disease severity, patients on therapy should be included to assess the effectiveness of therapy and alterations in the levels of CF mt-DNA.

## CONCLUSION

The present study is the first to elucidate the levels of CF mt-DNA in HIV, HBV and HCV infections among Pakistani population. Higher levels of CF mt-DNA were associated with viral infections. Elevated levels of CF mt-DNA did not co-relate with viral load in all the studied infections among the Pakistani population. This requires further investigation of the genetic variability of viral genomes which can be related to drug resistance and a possible link to increased inflammation resulting in the elevated levels of CT mt-DNA.

### Author’s Contribution

**MA, SW, ZA:** Conceptualization:

**ZA:** Investigation

**ZA, RAA, SW:** Formal Analysis and Manuscript writing:

**RAA, MA:** Resources

Mariam Anees is responsible and accountable for the accuracy and integrity of the work.
